# Array Designer: automated optimized array design for functional near-infrared spectroscopy

**DOI:** 10.1117/1.NPh.5.3.035010

**Published:** 2018-09-13

**Authors:** Sabrina Brigadoi, Domenico Salvagnin, Matteo Fischetti, Robert J. Cooper

**Affiliations:** a University College London, Biomedical Optics Research Laboratory, Department of Medical Physics and Biomedical Engineering, London, United Kingdom; b University of Padova, Department of Developmental Psychology, Padova, Italy; c University of Padova, Department of Information Engineering, Padova, Italy; d NeoLAB, Rosie Hospital, The Evelyn Perinatal Imaging Centre, Cambridge, United Kingdom

**Keywords:** functional near-infrared spectroscopy, array design, optode placement, probe design

## Abstract

The position of each source and detector “optode” on the scalp, and their relative separations, determines the sensitivity of each functional near-infrared spectroscopy (fNIRS) channel to the underlying cortex. As a result, selecting appropriate scalp locations for the available sources and detectors is critical to every fNIRS experiment. At present, it is standard practice for the user to undertake this task manually; to select what they believe are the best locations on the scalp to place their optodes so as to sample a given cortical region-of-interest (ROI). This process is difficult, time-consuming, and highly subjective. Here, we propose a tool, Array Designer, that is able to automatically design optimized fNIRS arrays given a user-defined ROI and certain features of the available fNIRS device. Critically, the Array Designer methodology is generalizable and will be applicable to almost any subject population or fNIRS device. We describe and validate the algorithmic methodology that underpins Array Designer by running multiple simulations of array design problems in a realistic anatomical model. We believe that Array Designer has the potential to end the need for manual array design, and in doing so save researchers time, improve fNIRS data quality, and promote standardization across the field.

## 1 Introduction

Functional near-infrared spectroscopy (fNIRS) is an optical technique that is used to monitor functional activation in superficial regions of the brain.^1^^,^^2^ Oxy- and deoxyhemoglobin are the principal absorbers of light in the near-infrared range, but human tissues are also relatively transparent to light at these wavelengths. Changes in the measured intensity of light that is emitted by a source on the scalp and backscattered to a detector placed nearby can be used to recover concentration changes of oxyhemoglobin (HbO) and deoxyhemoglobin (HbR) occurring in the superficial cortex.^3^ Each source and detector placed close enough to one another such that a measurement can be made is considered a “channel.” The depth into tissue to which a given channel is sensitive is a function of the distance between the source and detector on the scalp. Shorter distances (less than ∼10  mm) will result in channels that almost solely probe the extracerebral layers in the adult,^4^ whereas larger distances will demonstrate increasing relative sensitivity to the brain at the expense of a reduction in the signal-to-noise ratio (SNR) of the measured signal^5^ (simply because of the lower number of photons that will reach the detector). For the adult population, it has been shown that a good compromise between cortical sensitivity and SNR can be achieved with most fNIRS devices using a source–detector distance of ∼30  mm.^6^^,^^7^

Source and detector (or “optode”) positions on the scalp, and their relative separations, determine the sensitivity of each fNIRS channel to the underlying cortex.^8^ As a result, the exact positioning of optodes on the scalp is critical to every fNIRS experiment. It remains extremely rare for any fNIRS system to provide enough sources and detectors to adequately cover the whole scalp and thus sample the whole superficial cortex.^9^ Even where commercial systems provide the option of whole-scalp coverage, it is unusual to see fNIRS papers that employ the number of fibers required to achieve this,^10^ principally because of the significant ergonomic challenge of covering the full scalp with fiber optics. Instead, it is a standard practice for users to manually select what they believe are the best locations on the scalp to place their available optodes so as to sample the underlying cortical region-of-interest (ROI).

Solving this “array design problem” and creating a high-quality array for an fNIRS experiment is a complicated, multifactorial problem, which requires significant technical and neuroanatomical knowledge. It is the first, and arguably the most important, step in the preparation of an fNIRS experiment, and because of changes in the ROI and differences in head circumference at different ages, it usually has to be performed again for every new fNIRS study and for every new population.

The most common methodology for the design of fNIRS arrays is to exploit the scalp-brain correspondences computed for the standard EEG 10-20 reference system^11^ (or its higher density derivatives). The 10-20 positions can be used as a proxy for a given brain ROI, and fNIRS channels can be located around those positions. This methodology has previously been described in both adults^11^^,^^12^ and infants.^13^^,^^14^ Another approach is to employ a neuronavigation system to identify the scalp projection of a particular brain area of interest, identified by its MNI coordinates, and to position the midpoint of each channel over this projection. This method was generalized by Cutini et al.,^15^ who suggested the use of a physical model of the ICBM-152 MNI template for the neuronavigation procedure. Both of these methods are, however, highly subjective: once the scalp positions associated with a given brain ROI are identified, users must manually position their optodes to yield fNIRS channels that they think best align with the identified scalp locations. They also assume that centering an fNIRS channel over a point on the cortex will provide the highest sensitivity to that point, which is not necessarily true in a complex, multicompartment geometry like the human head.

The advent of photon transport simulations^16^^–^^18^ has yielded the possibility of calculating objective metrics of array quality, allowing users to quantify the sensitivity of a given channel to a specified cortical region. Several numerical methods exist to solve photon transport problems, such as the finite-element method or Monte Carlo approaches.^17^^,^^19^ These methods provide numerical solutions to the radiative transport equation (or the diffusion approximation to the radiative transport equation), which can be used to calculate the fluence distribution produced by a source transmitting light into a highly scattering medium. By taking the product of the source fluence distribution and the adjoint fluence distribution (that of a detector modeled as a source), the photon measurement density function (PMDF) can be calculated.^20^ The PMDF provides a model of the probability that a given photon transmitted from the source and measured at the detector has travelled through a given region of tissue. This is equivalent to a measurement of how sensitive a given fNIRS channel will be to a change in chromophore concentration in a given region of tissue. These PMDFs (or “sensitivity distributions”) provide an objective measure of the sensitivity (and therefore experimental utility) of a given fNIRS channel. Developments in hardware and software have resulted in a significant drop in the computational cost of these photon transport simulations, which can now be easily run on most standard computers.

One of the first attempts to exploit photon transport simulations to assist with the array design problem came with the implementation of AtlasViewer,^21^ a toolbox of the fNIRS processing package Homer2.^22^ With AtlasViewer, users can design an array by virtually locating optodes in almost any position on the scalp of an atlas (or an individual, MRI-derived) head model and map the sensitivity of that array on to the cortical surface. This approach provides an objective measure of an array’s sensitivity to a given cortical location. However, designing an array with AtlasViewer requires an iterative (and manual) approach: users design their array, run the photon migration simulations, map the sensitivity distribution on to the cortical layer, evaluate the result, and then amend their design and repeat the process until they adjudge the result to be adequate. This procedure was also the basis of the methodological paper by Wijeakumar et al.^23^ While providing meaningful and objective input, the AtlasViewer array design process remains subjective and can be time consuming. It is also worth noting that despite AtlasViewer’s functionality, no fNIRS study to date has (to our knowledge) reported any quantified measure of “array quality” as part of its experimental methods, which hinders interstudy comparisons.

The question that AtlasViewer seeks to answer is whether a given array design is sensitive or not to a specific area of the brain. However, the question a user wishes to answer is actually the exact inverse: given a brain ROI, what is the optimum array design?

The recently described fOLD toolbox, proposed by Zimeo Morais et al.,^24^ goes one step toward answering this question. This toolbox uses the PMDFs computed for a predefined grid-style array, allows users to define an ROI, and then outputs an ordered list of channels from its predefined array that demonstrate the highest sensitivity to that ROI. While easy to use, this toolbox operates in a highly constrained solution space (i.e., the array layout itself is already defined), and it is therefore not generalizable. It is also important to acknowledge that fNIRS array design is a combinatorial problem: determining an optimum array design for multiple sources and detectors is not simply a question of selecting the best individual channels. The array with the greatest sensitivity to a given brain ROI will not necessarily include the channel with the single greatest sensitivity to that ROI.

The first (and to our knowledge only) attempt to algorithmically solve the array design problem was that published by Machado et al.^25^ Their aim was to develop a method to compute an fNIRS array that provides the maximum sensitivity to specific brain regions that had been identified as the foci of epileptic discharges in adult epilepsy patients, and to do so using the fewest number of optodes. They employed a highly constrained (fNIRS system-specific) solution space that consisted of either an isometric arrangement with up to 123 potential optode positions or a subset of the 10-5 EEG system, containing up to 248 potential optode positions. In their paper, Machado et al.^25^ defined an optimum array as that which provides the highest total sensitivity to a target ROI. This objective was entirely appropriate for their stated goal, which was to optimally sample focal regions within the brains of their patients. However, although attempting to maximize the total sensitivity may seem like the correct approach to fNIRS array design, it does not generalize across the range of common array design problems.

Since 2012, we have been investigating how to design a tool that can provide fNIRS users with an optimized array design for their specific experiment, device, and population, in as unconstrained and easily applicable a manner as possible. A prototype of this tool was presented at the fNIRS 2016 conference in Paris. In this paper, we describe and validate the algorithmic methodology that underpins our tool: Array Designer, which we hope will end the need for manual array design, and in doing so save researchers time, improve fNIRS data quality, and promote standardization across the field.

## 2 Materials and Methods

### 2.1 Problem Formulation and Data Preparation

Solving the array design problem requires an anatomically appropriate head model and a range of potential optode positions (i.e., a solution space), which must relate to a reference system that allows users to transfer their solution from software into the physical world. It also requires the PMDFs of all possible viable channels (or means of creating them). In addition, there are a series of variables related to the specific array design problem. These include the user-defined ROI, how many sources and detectors are available, and what source–detector separations are viable for the user’s fNIRS system. In the following sections, we describe how we formulated the problem and prepared the necessary data. We also outline the objective function that Array Designer is designed to maximize.

#### 2.1.1 Anatomical head model

The optimum array should be calculated in a head model that is as similar as possible to the population under investigation or even within a subject-specific head model, if appropriate (e.g., for clinical applications). Several head models have been recently proposed that span the neonatal^26^ and adult populations.^4^^,^^21^ It is our goal to ultimately allow each user to select the best head model (or individual head model) for their study since the methods proposed here are generalizable to any head model. However, for testing and validation purposes, we decided to employ the adult head model built from the nonlinear asymmetric MNI-ICBM152 atlas.^27^ This head model was built as described in Brigadoi and Cooper^4^ and Dempsey et al.^28^ and can be freely downloaded at Ref. 29. This package contains high-density scalp surface, gray matter (GM) surface, and head volume meshes. Note that for the purposes of testing Array Designer, the original GM surface mesh (number of nodes: 30327) was downsampled by a factor of 2.5 using the iso2mesh toolbox^30^ to yield a surface mesh of 12,038 nodes. The downsampled GM mesh preserved the critical anatomical details (i.e., the cortical folding) but allowed us to reduce the computational time required by some of the algorithms we tested.

#### 2.1.2 10-2.5 reference system

**Fig. 1:**
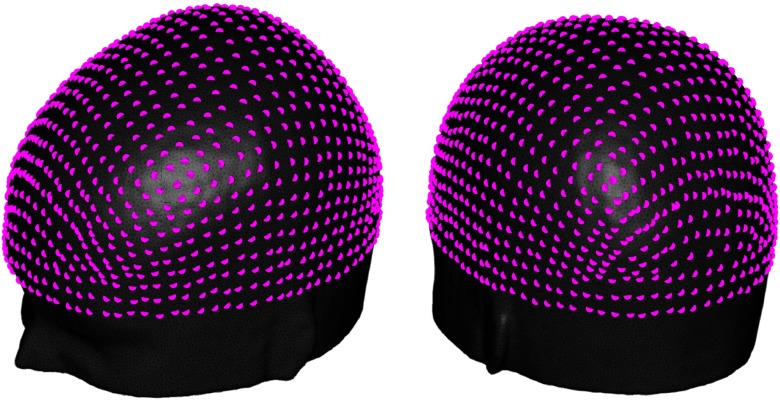
10-2.5 system. In magenta, all points of the 10-2.5 system, our solution space, are visualized over the adult MNI152 scalp surface mesh.

Users often employ the 10-5 EEG system,^31^ which comprises 345 points, as a reference system when positioning their optodes, i.e., as their solution space. However, the distance between nearest-neighbor points in the 10-5 system averages 17±1.9  mm, and consequently, the second nearest neighboring points often exceed 30 mm. As a result, the density of the 10-5 system is not ideal for fNIRS array design, as arrays typically include channels between 20 and 50 mm in the adult brain,^32^ usually with an optimum distance of 30 to 35 mm. Using the 10-5 system therefore inevitably reduces our ability to position the optodes in an optimum way. In order to increase the solution space, we computed an increased density version of the 10-5 system: the 10-2.5 system, using the scalp surface mesh introduced above. This system comprises 1092 points and was built by adding points halfway between two existing 10-5 locations, computed according to Oostenveld and Praamstra’s definition^31^ (see Fig. 1). The average distance between neighboring points in this system is 8.7±1  mm, which increases the number of positions that can be paired to yield valuable fNIRS channels. Of course, it is possible to increase the density of the solution space arbitrarily, for example, to a 10-1.25 system. However, these higher density systems will be difficult to translate in practice since the distance between neighboring points will likely be less than the precision with which a cap can be prepared and applied by the experimenter.

#### 2.1.3 PMDF computation

Photon migration simulations were performed with the TOAST++ software^18^ for each of the 10-2.5 points within MATLAB (Mathworks, Massachusetts). These simulations only need to be run once for each head model and can then be stored as described below for repeated use by Array Designer. Using the MNI-152 head volume mesh, optical properties were assigned to each tissue type (scalp, skull, cerebrospinal fluid, gray and white matter) by fitting all published adult values across the NIR spectrum and selecting the fitted values at a wavelength of 800 nm.^33^^–^^35^ Fluence distributions were then computed for each 10-2.5 position in the volume mesh. To minimize computational burden, rather than computing every PMDF in the high-density volume mesh, the volumetric fluence distributions for each 10-2.5 point were first projected on to the GM surface mesh. We then computed GM surface-based PMDFs for all viable channels using the adjoint method. Viable channels were defined as any possible pair of 10-2.5 points within a maximum distance of 60 mm of one another (77,995 channels). The PMDFs were calculated for a given channel by taking the product of the GM-surface fluence distributions for a selected source and detector, correcting for elemental mesh volumes and normalizing by the value of the volumetric source fluence distribution at the detector position or the value of the volumetric detector fluence distribution at the source position (whichever is larger). This normalization factor is referred to as ${\mathrm{PMDF}}_{\text{norm}}$. All PMDF values smaller than $1\times 10^{-6}$ times the maximum value of the PMDF were set to zero to promote the sparsity of the matrix. The resulting GM surface distribution vectors each had dimensions of 1×12,038 (number of GM nodes). A total of 77,995 sparse surface PMDF matrices were calculated and stored in a MATLAB cell array of dimensions 1092×1092, each cell being a possible combination between two 10-2.5 points. When saved to the disk, the full PMDF data occupied 1.79 Gb of hard drive space.

As mentioned above, the relative sensitivity of an fNIRS channel to the brain increases with source–detector separation^6^ while the measured intensity of light (and thus the SNR) decreases. If one does not account for this decrease in SNR, any array optimization method that seeks to maximize sensitivity will always attempt to maximize source–detector separations, which will have little practical utility. It was therefore necessary to design an approach that allows the PMDFs calculated in the above simulations to be weighted to account for the dynamic range of the fNIRS system of a given user, and thus account for this critical SNR consideration. To achieve this, Array Designer computes a weighting factor that is applied to each PMDF. This weighting factor is computed on the basis of two user-defined inputs. These are the maximum permissible source–detector separation (*maxRho*) and the source–detector separation that the user considers the best balance between maximizing source–detector separation and SNR (*maxGoodRho*). The idea of the weighting factor is to penalize channels that have a greater separation than *maxGoodRho*, and to set to zero the PMDFs associated with source–detector separation channels that exceed *maxRho*. To determine these weighting factors, we first compute the ratio of the ${\mathrm{PMDF}}_{\text{norm}}$ for all channels with a separation greater than *maxGoodRho* to the average ${\mathrm{PMDF}}_{\text{norm}}$ value for all channels with a separation equal to *maxGoodRho*. This calculation yields values that decay exponentially as source–detector separation increases beyond *maxGoodRho*. We then computed a linear fit to the logarithm of these values as a function of source–detector separation. Last, the weighting function for a given source–detector distance (${\mathrm{SD}}_{i}$) was calculated by taking the exponential of the slope coefficient of this linear fit (a, which is negative), multiplied by the difference between ${\mathrm{SD}}_{i}$ and *maxGoodRho*. The weighting factor ($W_{\mathrm{PMDF}}$) can thus be defined as follows:

$$W_{\mathrm{PMDF}}(i)=\{\begin{array}{ll} 1 & {\mathrm{SD}}_{i}\leq \mathrm{maxGoodRho}\\ e^{a*({\mathrm{SD}}_{i}-\mathrm{maxGoodRho})} & \mathrm{maxGoodRho}<{\mathrm{SD}}_{i}\leq \mathrm{maxRho}\\ 0 & {\mathrm{SD}}_{i}>\mathrm{maxRho} \end{array}$$

**Fig. 2:**
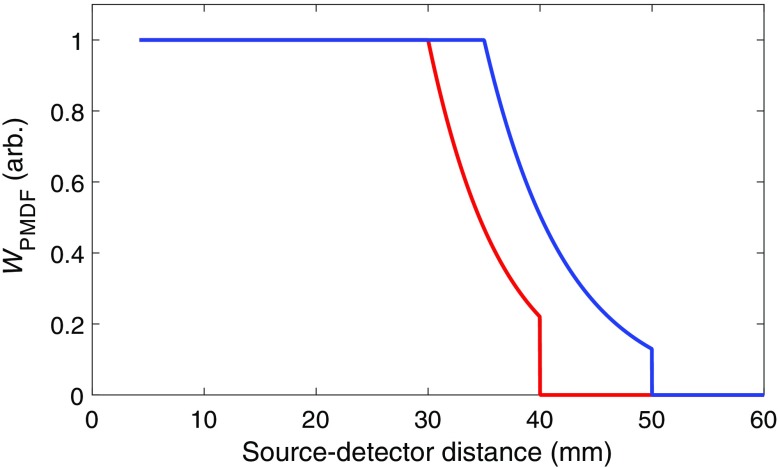
Examples of the weighting factor $W_{\mathrm{PMDF}}$, as a function of source–detector separation. The red line shows $W_{\mathrm{PMDF}}$ for a *maxGoodRho* set to 30 mm and a *maxRho* of 40 mm. For the blue line, maxGoodRho=35  mm and maxRho=50  mm. $W_{\mathrm{PMDF}}$ is always equal to 1 for all PMDFs with source–detector separations smaller than *maxGoodRho* and zero for all PMDFs with source–detector separations greater than *maxRho*. Note that the smallest possible source–detector distance is 4.2 mm, which is the smallest distance between any two 10-2.5 points.

Figure 2 shows the weighting factor $W_{\mathrm{PMDF}}$ as a function of source–detector separation for two different values of *maxGoodRho* and *maxRho*.

#### 2.1.4 User inputs

Array Designer requires certain inputs to allow the problem to be defined. While we have designed our methods to be generalizable, such that in the future, it will be possible for the user to select their preferred head-model, at present, we consider the head model and the unweighted PMDFs to be fixed. The remaining user-settable parameters that together define each array design problem are then as follows:

*nS* is the number of source optodes available;
*nD* is the number of detector optodes available;
*minRho* is the minimum source–detector distance allowed for a viable channel;
*minRhoOpt* is the minimum physical distance allowed between any two optodes;
*maxGoodRho* is the maximum source–detector distance that reliably produces data with a good SNR;
*maxRho* is the maximum source–detector distance allowed for a viable channel;
*ROI* is a binary mask in the GM surface space that defines the areas of the cortex under investigation.

Note that *minRho* and *minRhoOpt* are both required: *minRhoOpt* can be thought of as the physical size of the optodes and is thus the closest any two optodes can be placed. This must be independent of the minimum channel separation (*minRho*), primarily because of the issue of detector saturation. For example, if one has 10-mm diameter optodes, but a detector may become saturated when <20  mm from a source, a good array design algorithm must have the option to place two or more detectors at between 10 and 20 mm from one another, and/or two or more sources at 10-20 mm from one another while simultaneously ensuring no source is less than 20 mm from any detector.

#### 2.1.5 Objective function

In their pioneering work, Machado et al.^25^ chose to maximize the total sensitivity of an array to the ROI. That is to say, their objective function was simply the sum of all the PMDF values that fall inside the ROI, over all channels within an array. Machado et al.^25^ also failed to account for the system SNR, as described above, meaning that their algorithm likely sought to maximize source–detector separation wherever possible. This approach was appropriate for their purpose, which was to cover small, focal ROIs in patients with epilepsy using a relatively constrained solution space. However, it is not ideal for general fNIRS applications. Maximizing the total sensitivity will tend to encourage the clustering of channels over the location within the ROI that is closest to the surface. If the ROI is focal, this is the optimum result. However, if the ROI is spatially extended (as is usually the case in fNIRS applications), maximizing the sensitivity alone will yield inappropriate results.

Maximizing the mean or the median of the sensitivity distribution over the ROI also yields suboptimal results. Maximizing the mean value produces identical results as when maximizing the total sensitivity (as one is simply dividing the objective function by a constant). The same is effectively true of maximizing the median sensitivity since any ROI that contains both shallow and deep nodes (i.e., over gyri and sulci), will always yield a distribution of sensitivities that is highly skewed. As a result, an algorithm attempting to maximize the median sensitivity over the ROI will tend to continue to increase the sensitivity to the shallowest nodes and skew the distribution upward as far as possible.

Ideally, an optimum array should have two concurrent features: (1) it should provide the highest sensitivity to the given ROI and (2) it should cover as much of the ROI as possible. We therefore developed an objective function that accounts for both of these features. We define our objective function as follows:

$$\arg\;\underset{A}{\max}[S_{A}+cW*C_{A}],$$ (1)

where

$$S_{A}=\frac{\sum_{n\in \mathrm{ROI}}{\mathrm{PMDF}}_{i}(n)}{S_{\max}}$$ (2)

and

$$C_{A}=\frac{N_{\mathrm{thresh}}}{N_{\mathrm{tot}}}.$$ (3)

Here, $S_{A}$ is the normalized total sensitivity and $C_{A}$ is the normalized coverage of the ROI provided by array A. The component $S_{A}$ is computed as the sum over ROI nodes of the sensitivity distribution of the selected set of channels (i), divided by the sum over ROI nodes of the maximum sensitivity achieved when maximizing the $S_{A}$ term only ($S_{\max}$), as was performed by Machado et al.^25^ The component $S_{A}$ will therefore always assume values between 0 and 1, where 1 corresponds to an array with a sensitivity equal to that achieved when sensitivity is the only consideration.

The component $C_{A}$ was computed as the ratio between $N_{\mathrm{thresh}}$ (the number of nodes of the ROI with a total sensitivity exceeding a predefined coverage threshold $C_{\mathrm{thresh}}$) and $N_{\mathrm{tot}}$, the total number of nodes within the ROI. The user-defined parameter coverage weight (*cW*) allows the importance of the coverage component of the objective function to be selected. Setting *cW* to zero is equivalent to maximizing the total array sensitivity only, with no consideration of coverage. Setting *cW* to a nonzero positive value will force the algorithm to simultaneously maximize the proportion of the ROI that exceeds a minimum sensitivity and is therefore considered “covered.” This has the effect of forcing the array to spread out over the ROI. The coverage threshold ($C_{\mathrm{thresh}}$) was defined as follows:

Cthresh=log(100+pthresh100)actvolV^*Δμa,

where $p_{\mathrm{thresh}}$ is the measured change in intensity signal that users should expect to be measurable with their system (e.g., 1%), ${\mathrm{act}}_{\mathrm{vol}}$ is an approximate volume over which a hemodynamic response can be expected to occur, V^ is the median Voronoi volume across nodes of the GM mesh and $\Delta \mu_{a}$ is the approximate change in absorption coefficient expected during a hemodynamic response. The coverage threshold defines when a node can be considered as covered by a given array. Here, we considered a node to be covered when a change in $\Delta \mu_{a}$ by 10% ($∼0.001\;{\mathrm{mm}}^{-1}$) in a block of tissue of $1\;{\mathrm{cm}}^{3}$ (${\mathrm{act}}_{\mathrm{vol}}$) will yield a change in intensity in the measured fNIRS signal larger than 1% ($p_{\mathrm{thresh}}$).

### 2.2 Array Designer Algorithm: GRASP

The greedy randomized adaptive search procedure (GRASP)^36^^,^^37^ is a metaheuristic algorithm commonly applied to combinatorial optimization problems. GRASP is an iterative process: at each iteration, a greedy randomized solution is constructed and tentatively improved through local search. Each greedy randomized solution is constructed incrementally by adding elements (optodes) to the current array solution from a list of candidates ranked by a greedy function. To obtain variability, the best candidate elements are put into a restricted candidate list, from which one is chosen at random.

Here, we implemented two GRASP metaheuristics: the first for the simpler case of maximizing the total sensitivity, which was only needed to allow normalization of the sensitivity term in the objective function of Eq. (1); and the second for maximizing both the normalized total sensitivity and the normalized coverage of the ROI.

GRASP begins with a construction heuristic and an empty solution, in which no optode is placed. The algorithm then ranks all possible source–detector pairs according to their contribution to the total sensitivity and chooses one randomly among the top five pairs. GRASP then iteratively tries to add a source or a detector (alternating between the two). The objective contribution of adding a source (detector) in each of the available positions is computed (using the objective function with only the sensitivity term or that containing both $S_{A}$ and $C_{A}$ terms, depending on the case), and the positions are ranked accordingly. A source (detector) is then added to the array solution by randomly selecting one from the top five ranked positions. Note that after adding each source/detector, the set of candidate positions is updated to take in to account *minRho*, *minRhoOpt*, and *maxRho*. This process is then iterated until the required numbers of sources and detectors are placed.

Once a complete solution has been constructed, GRASP tries to improve upon it via a local search. For both objectives of interest, a 1-opt neighborhood is applied first, in which we remove a single source (or detector) and try to find a better position for it while keeping all other optodes in place. Again, when adding or removing an optode, the set of positions that are feasible is updated to account for the distance constraints. If the current solution is locally optimal according to this neighborhood, a more expensive neighborhood is then searched. In the simpler case of maximization of just the total sensitivity, a flip-float neighborhood, as described by Glover et al.^38^ for the bipartite Boolean quadratic programs problem, was implemented. The idea is that for a fixed set of sources (or detectors), the optimal set of detectors (sources) can be easily computed in a greedy fashion (technically, this is true only if there are no incompatibilities or distance constraints: however, the logic can be heuristically extended to take these additional constraints into account). Thus, the neighborhood is defined by removing one source (detector), testing each possible new position, and recomputing the optimal set of detectors (sources) in each case. For the more complicated objective function [Eq. (1)], it is no longer true that for a fixed set of sources, the set of optimal detectors can be greedily computed (even if we ignore the distance constraints). The flip-float neighborhood therefore cannot be implemented efficiently. In this case, a more traditional 2-opt neighborhood was implemented, where at each iteration, a source–detector pair is removed and the algorithm tries to find a better position for the two optodes simultaneously.

### 2.3 Comparison with Manual, Single-Distance Arrays

In order to determine the efficacy of our proposed solution to the array design problem, it is necessary to compare our results with those achieved by a traditional, manual approach. To provide this comparison, we chose to create single-distance (i.e., alternating grid-style) arrays that approximate the arrays that are still used by many fNIRS systems. First, we found the center-of-mass of each ROI. We then determined the 10-2.5 positions that yielded an array that was positioned over the ROI center-of-mass and which had each first-nearest-neighbor source–detector distance as close as possible to 30 mm. A single-distance array was manually constructed in this fashion for each of the simulated array design problems described in Sec. 2.5. Note that these manual arrays were built to emulate standard single-distance arrays. When nS and nD are large (or approximately equal), these arrays tend to form alternating grids. However, when nS≪nD, these arrays were built to simulate a star pattern with a central source (or sources) surrounded by many detectors.

### 2.4 Algorithms for Comparison

In order to determine the efficacy of our proposed solution to the array design problem, it is also clearly necessary to compare our results with those achieved by the prior published attempt to algorithmically solve the array design problem, i.e., that of Machado et al.^25^ However, Machado’s mixed integer problem (MIP) optimization method is incompatible with the simultaneous maximization of sensitivity and coverage. As a result, we decided to validate our approach as follows: first, we would recreate Machado’s MIP algorithm (${\mathrm{MIP}}_{\mathrm{MACH}}$), with the same objective function and formulation, as described in their paper (see Sec. 2.4.1). We would then create a new MIP formulation that follows similar optimization methodology but enables the simultaneous maximization of sensitivity and coverage (${\mathrm{MIP}}_{\mathrm{NEW}}$—see Sec. 2.4.2). This new MIP formulation would then be tested and validated to ensure that it performs comparably to Machado’s formulation when coverage is ignored. In this comparison, the objective function used by ${\mathrm{MIP}}_{\mathrm{MACH}}$ and ${\mathrm{MIP}}_{\mathrm{NEW}}$ will be exactly the same, whereas the MIP formulation will be different. Having tested the efficacy of ${\mathrm{MIP}}_{\mathrm{NEW}}$ when coverage is ignored, the solutions generated by this new MIP formulation will be compared with ${\mathrm{MIP}}_{\mathrm{MACH}}$ when ${\mathrm{MIP}}_{\mathrm{NEW}}$ is set to optimize for both sensitivity and coverage. This comparison should demonstrate the advantages of employing this combined objective function. Once these steps are complete, ${\mathrm{MIP}}_{\mathrm{NEW}}$ can appropriately be used as a benchmark against which to test Array Designer’s GRASP algorithm.

MIP is a well-established paradigm for mathematical optimization problems. An arbitrary MIP problem is defined by a finite number of variables, some of which are constrained to take only integer variables, a finite number of linear constraints, and a linear objective function. While constrained at first sight, the paradigm is flexible enough to encode a lot of optimization problems of theoretical and practical interest. More importantly, the paradigm is now a mature technology, and many general-purpose MIP solvers are available, both commercial and open-source. One of the advantages of the MIP paradigm is the availability of bounds on the optimum objective value. These bounds provide a measure of how far a given solution is from optimal.

#### 2.4.1 Machado MIP formulation

The mathematical formulation of ${\mathrm{MIP}}_{\mathrm{MACH}}$ is reported in Appendix. This model maximizes the total sensitivity only and exploits the fact that the total sensitivity is a linear function. Furthermore, it is not necessary for this model to compute the sensitivity at each individual node in the ROI, as it aims to maximize only the sum of the sensitivity over all the nodes within the ROI. As a result, this sum can be precomputed. Note that the system SNR consideration described in Sec. 2.1.3 above is accounted for by the weighting of the PMDFs (i.e., it is solved by amending the data parsed to each algorithm rather than amending the algorithms themselves). As a result, while ${\mathrm{MIP}}_{\mathrm{MACH}}$ employs the exact algorithm described by Machado et al.,^25^ our application of it here will yield arrays that do not simply pursue maximized source–detector separations and will thus provide a better comparator for our algorithms.

#### 2.4.2 New MIP formulation

The model ${\mathrm{MIP}}_{\mathrm{MACH}}$ cannot be easily extended to take the coverage part of the objective function into account. For this reason, a different formulation was devised. The mathematical formulation of ${\mathrm{MIP}}_{\mathrm{NEW}}$ is reported in Appendix. In this new model, the number of variables is quadratic in the number of possible positions at which to place an optode. Other two variables, one binary encoding the coverage part of the objective and one continuous encoding the sensitivity part of the objective, are required for each node of the ROI. Since this new model aims to maximize both sensitivity and coverage, it is necessary to compute and record the sensitivity at each individual node in the ROI. As a result, this model is expected to be less efficient than ${\mathrm{MIP}}_{\mathrm{MACH}}$.

### 2.5 Simulations and Validation Methods

The performance of the GRASP algorithm, of the two MIP algorithms, and of the manual single-distance arrays was tested and compared by running a range of array design simulations. The simulation parameters comprised the ROI, the number of sources (*nS*) and detectors (*nD*), and the *cW* value. To minimize the number of necessary simulations, the following parameters were kept fixed in all simulations, which we felt was reasonable given the results of altering these parameters are highly predictable: *minRho* (15 mm), *minRhoOpt* (10 mm), *maxRho* (60 mm), and *maxGoodRho* (30 mm). $C_{\mathrm{thresh}}$ was computed to be equal to 0.1528 mm for our downsampled GM mesh.

**Fig. 3:**
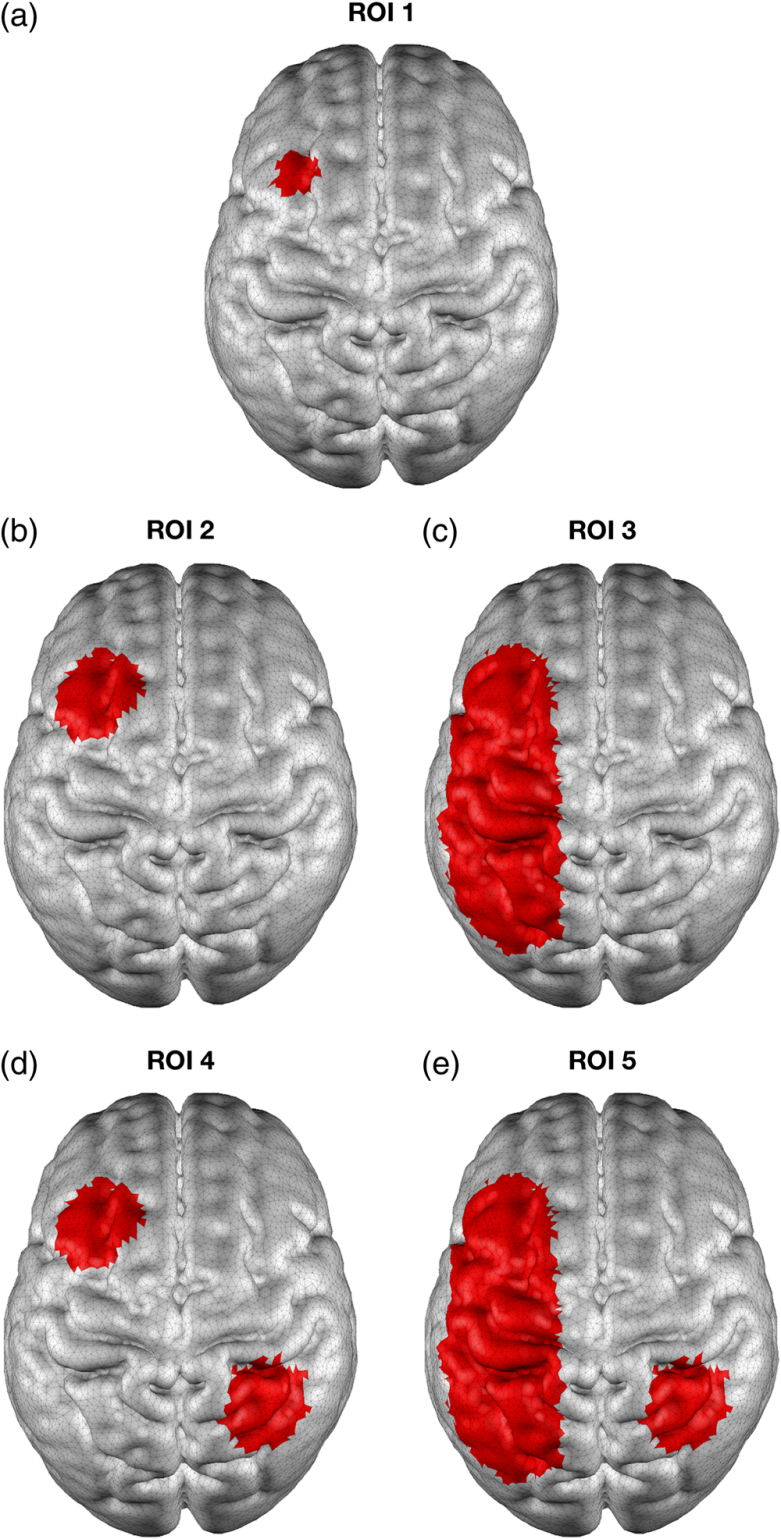
The five example ROIs used to test the performance of each approach: (a) ROI 1, (b) ROI 2, (c) ROI 3, (d) ROI 4, and (e) ROI 5.

Five example ROIs were defined on the GM surface mesh. These five ROIs varied in shape and extent, so as to fully test the performance of each algorithm in a range of realistic scenarios (see Fig. 3): ROI 1 was based on a small single spherical region with a 10 mm radius over the left frontal cortex; ROI 2 on a single spherical region with a 20 mm radius over the left frontal cortex; ROI 3 on an extended 3-D ellipsoid region over the left frontal, motor and parietal cortex; ROI 4 on two distinct spherical regions, each with a 20 mm radius, one located in the left frontal cortex and the other in the right parietal cortex; and ROI 5 was based on a small spherical region with a 20 mm radius located in the right parietal cortex and an extended 3-D ellipsoid region over the left frontal, motor and parietal cortex. All ROIs were defined by finding the GM surface nodes that are contained within these volumetric shapes. As a result, each ROI incorporates both gyri and sulci.

The number of sources and detectors was simulated to be 1, 2, 4, 8, or 16, with nD≥nS in all cases, under the reciprocity assumption that the same solution should be obtained when using n sources and m detectors or m sources and n detectors. The *cW* parameter, which is only relevant for the GRASP and ${\mathrm{MIP}}_{\mathrm{NEW}}$ formulations, assumed one of the following values: 0, 1, 2, 3, 5, and 10. For the GRASP and our MIP formulation, therefore, a total of 450 different simulations were performed (5 ROIs×15 source–detector combinations × 6 *cW* values), while for the original MIP formulation and the manual single-distance arrays, a total of 75 simulations were performed (5 ROIs×15 source–detector combinations). Each algorithm was given a maximum of 1 h to find a solution in each simulation. Each MIP simulation was performed using the IBM CPLEX Optimizer software 12.7.1^39^ on a Linux desktop with Intel Core i5 750 running at 2.67 GHz and 8 Gb of RAM. Each GRASP simulation was performed on a standard 2014 Apple MacBook Pro with 8 Gb of RAM.

Four metrics were employed to compare the results of these simulations: the time required by the algorithm to converge (which is obviously not available for the manual single-distance arrays), the total sensitivity of the resulting array to the ROI, the ratio of the total sensitivity of an array to the maximum total sensitivity achieved by any algorithm or method, and the percentage of ROI nodes covered by the array.

## 3 Results

### 3.1 Comparison of Manual Single-Distance Arrays and Array Designer

**Fig. 4:**
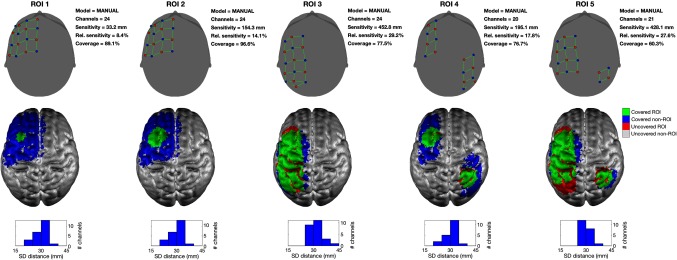
Solutions obtained with the manual, single-distance arrays. In this example, the arrays obtained manually for all five ROIs and nS=nD=8 are depicted. In the upper row, sources are shown as red circles, detectors as blue circles, and channels in green. For each array, the number of viable channels, the absolute sensitivity of the array over the ROI, the relative sensitivity of the array over the ROI, and the relative coverage of the array are reported. The middle row displays the results on the GM surface. The area of the ROI covered by each array is shown in green, while any portion of an ROI that remains uncovered is shown in red. Brain areas that are covered but are outside of the ROI are shown in blue. The bottom row provides a histogram of the source–detector distances present within each array.

**Fig. 5:**
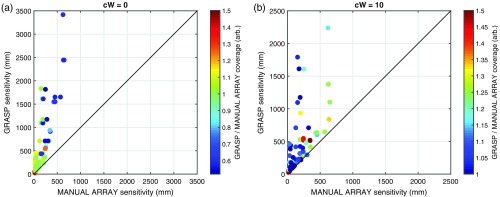
Comparison between manual single-distance arrays and Array Designer solutions with (a) cW=0 and (b) cW=10. The total array sensitivity of each solution achieved by the Array Designer approach (on the y axis) is plotted against that achieved by the manual single-distance array approach for 75 simulations. Each dot (representing one simulation) is color-coded based on the ratio of the coverage of the solution achieved by Array Designer to that obtained by the manual single-distance array. Note that the sensitivity achieved by Array Designer is always higher than that achieved by the manual single-distance arrays. For cW=10, both the sensitivity and the coverage achieved by Array Designer are always higher than that achieved by the manual single-distance arrays.

Figure 4 shows examples of the manually constructed, single-distance arrays in all five simulated ROIs. Figure 5 shows a scatter plot of the solution sensitivity for all the manual single-distance array simulations against the solution sensitivity achieved by Array Designer at two example *cWs* (cW=0 on the left and cW=10 on the right). Each point in the scatter plot is color-coded by the ratio of the coverage obtained by the two approaches. Figure 5 shows that Array Designer is always able to achieve considerably better sensitivity than the manual single-distance arrays for all *cWs*. When coverage is not strongly optimized (i.e., when *cW* is small), manual single-distance arrays tend to achieve higher coverage values compared to Array Designer in large ROIs but at the expense of a lower sensitivity.

### 3.2 Comparison of ${\mathrm{MIP}}_{\mathrm{MACH}}$ and ${\mathrm{MIP}}_{\mathrm{NEW}}$ Approaches

**Fig. 6:**
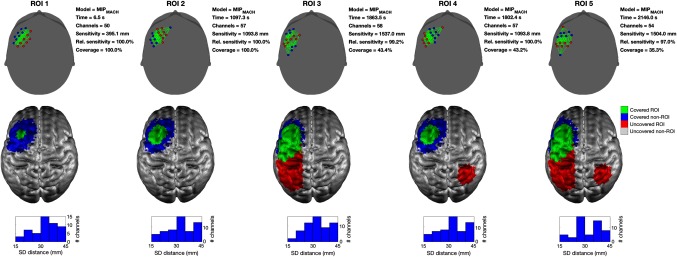
Solutions obtained with ${\mathrm{MIP}}_{\mathrm{MACH}}$. In this example, the arrays obtained by solving the array design problem with the algorithm of Machado et al. (Ref. 25) for all five ROIs and nS=nD=8 are depicted. In the upper row, sources are shown as red circles, detectors as blue circles, and channels in green. For each array, the time taken, the number of viable channels, the absolute sensitivity of the array over the ROI, the relative sensitivity of the array over the ROI, and the relative coverage of the array are reported. The middle row displays the results on the GM surface. The area of the ROI covered by each array is shown in green, while any portion of an ROI that remains uncovered is shown in red. Brain areas that are covered but are outside of the ROI are shown in blue. The bottom row provides a histogram of the source–detector distances present within each array.

As described above, the first step in the algorithmic validation of our solution to the array design problem was to compare the MIP approaches proposed by Machado et al.^25^ (${\mathrm{MIP}}_{\mathrm{MACH}}$), with the new formulation of the MIP approach (${\mathrm{MIP}}_{\mathrm{NEW}}$) that is able to simultaneously maximize both sensitivity and coverage. To highlight the necessity of this, Fig. 6 shows examples of the solutions obtained with ${\mathrm{MIP}}_{\mathrm{MACH}}$ in all five simulated ROIs. In the cases with extended ROIs (3, 4, and 5), even when using a relatively high number of sources and detectors (eight sources and eight detectors), the algorithm promotes a focused array solution that covers an area where the highest sensitivity can be achieved (i.e., where the cortex is closest to the scalp), rather than covering the whole ROI. When the ROI consists of multiple, noncontiguous regions, ${\mathrm{MIP}}_{\mathrm{MACH}}$ tends to cover only one of them (whichever is the more superficial). In the example shown in Fig. 6, it can be seen that ${\mathrm{MIP}}_{\mathrm{MACH}}$ produces very similar results for each ROI, irrelevant of its shape or extent. Note that the most common source–detector distance in each solution is close to the value of *maxGoodRho* (30 mm), which illustrates the impact of the SNR weighting function described in Sec. 2.1.3.

#### 3.2.1 Comparison of ${\mathrm{MIP}}_{\mathrm{MACH}}$ and ${\mathrm{MIP}}_{\mathrm{NEW}}$ with cW=0

**Fig. 7:**
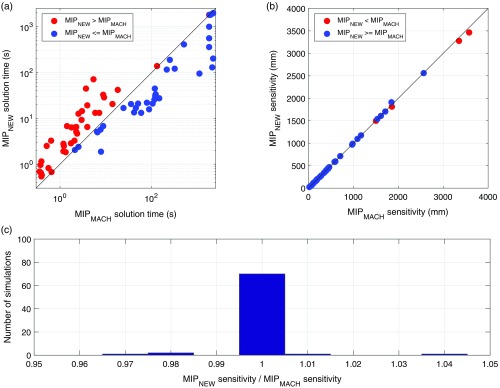
Comparison between the two MIP approaches. (a) A scatter plot comparing the solution times of the two MIP approaches. Values above the y=x line (red) correspond to simulations, where ${\mathrm{MIP}}_{\mathrm{NEW}}$ took longer than ${\mathrm{MIP}}_{\mathrm{MACH}}$. (b) A scatter plot comparing the total array sensitivity between the two MIP approaches. (c) A histogram of the ratio of the absolute sensitivity of the array over the ROI between the two MIP approaches.

This comparison is designed to examine whether the two MIP formulations provide different solutions when applied to exactly the same problem, with the same objective function. The results of this comparison are depicted in Fig. 7. The two MIP formulations take comparable periods of time to converge. Our new MIP formulation ${\mathrm{MIP}}_{\mathrm{NEW}}$ tends to take longer to reach a solution compared to ${\mathrm{MIP}}_{\mathrm{MACH}}$ when the solution can be reached quite quickly (within 20 s). When the solution takes longer to find, ${\mathrm{MIP}}_{\mathrm{NEW}}$ tends to be faster than ${\mathrm{MIP}}_{\mathrm{MACH}}$. In terms of the total sensitivity of the arrays created by the two MIP formulations, they achieve identical results in 70 of the 75 simulations [Fig. 7(c)]. The largest observed relative deviation in array sensitivity between the ${\mathrm{MIP}}_{\mathrm{NEW}}$ and ${\mathrm{MIP}}_{\mathrm{MACH}}$ solutions was 3.7%.

#### 3.2.2 Comparison of ${\mathrm{MIP}}_{\mathrm{MACH}}$ and ${\mathrm{MIP}}_{\mathrm{NEW}}$ with cW>0

**Fig. 8:**
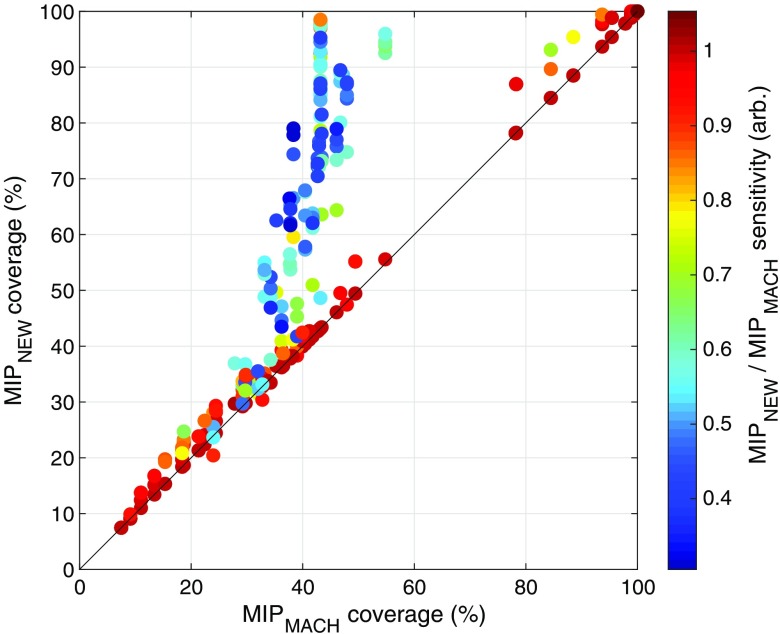
Comparison between the two MIP approaches, with cW>0 for ${\mathrm{MIP}}_{\mathrm{NEW}}$. The percentage coverage of each solution achieved by the ${\mathrm{MIP}}_{\mathrm{NEW}}$ approach (on the y axis) is plotted against the equivalent achieved by the ${\mathrm{MIP}}_{\mathrm{MACH}}$ approach for 450 simulations. For a given ROI, *nS* and *nD*, the solution achieved by the ${\mathrm{MIP}}_{\mathrm{MACH}}$ approach at different *cWs* will be always the same, while the solution obtained by ${\mathrm{MIP}}_{\mathrm{NEW}}$ will vary. Each dot (representing one simulation) is color-coded based on the ratio of the total sensitivity of the solution achieved by ${\mathrm{MIP}}_{\mathrm{NEW}}$ to that obtained by ${\mathrm{MIP}}_{\mathrm{MACH}}$. Note that the sensitivity achieved by ${\mathrm{MIP}}_{\mathrm{NEW}}$ is almost always comparable to that achieved by ${\mathrm{MIP}}_{\mathrm{MACH}}$ except in cases, where the new algorithm has improved coverage (the blue streak of points). The cluster of simulations where greater than ∼75% coverage was achieved with both MIP formulations corresponds to ROIs 1 and 2, which are focal and thus easy to cover.

This comparison is intended to highlight the difference between the solutions achieved when employing the objective function designed by Machado et al.^25^ and those obtained using our objective function that takes into account both the sensitivity and coverage of the ROI. Figure 8 shows a scatter plot of the solution coverage for all ${\mathrm{MIP}}_{\mathrm{NEW}}$ simulations (at all *cW*) against the solution coverage achieved by ${\mathrm{MIP}}_{\mathrm{MACH}}$. Each point in the scatter plot is color-coded by the ratio of the sensitivities obtained by the two MIP formulations. Figure 8 shows that ${\mathrm{MIP}}_{\mathrm{NEW}}$ is able to achieve significantly better coverage than ${\mathrm{MIP}}_{\mathrm{MACH}}$ but at the expense of total sensitivity.

### 3.3 Comparison between ${\mathrm{MIP}}_{\mathrm{NEW}}$ and Array Designer (GRASP)

**Fig. 9:**
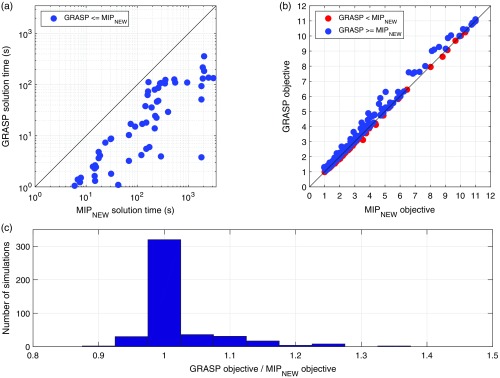
Comparison between Array Designer (GRASP) and the ${\mathrm{MIP}}_{\mathrm{NEW}}$ approach. (a) Comparison between the two approaches in terms of the time required by each algorithm to converge. (b) Comparison between the two approaches in terms of the value of the objective function obtained in each simulation. (c) A histogram of the ratio of the values of the objective function obtained between the two approaches. Note the histogram is skewed in a manner indicating the superior performance of Array Designer relative to the MIP formulation.

Having demonstrated that ${\mathrm{MIP}}_{\mathrm{NEW}}$ performs almost identically to ${\mathrm{MIP}}_{\mathrm{MACH}}$ when cW=0 and is able to improve coverage, we were able to use ${\mathrm{MIP}}_{\mathrm{NEW}}$ as a benchmark against which to validate the Array Designer (GRASP) approach for simulations with any *cW* value. Figure 9 provides a comparison between Array Designer and ${\mathrm{MIP}}_{\mathrm{NEW}}$ for all simulations. Figure 9(a) demonstrates that the GRASP algorithm is dramatically faster than ${\mathrm{MIP}}_{\mathrm{NEW}}$, always converging in less than 350 s. The Array Designer approach also provides objective function values that are greater than or equal to that of ${\mathrm{MIP}}_{\mathrm{NEW}}$ in 318 of the 450 simulations and very similar to ${\mathrm{MIP}}_{\mathrm{NEW}}$ in the remaining 132 simulations [Figs. 9(b) and 9(c)]. In the worst case, Array Designer produced a solution with an objective function value equal to 88% that of ${\mathrm{MIP}}_{\mathrm{NEW}}$, whereas in the best case, it achieved a value 136% that of the ${\mathrm{MIP}}_{\mathrm{NEW}}$.

**Fig. 10:**
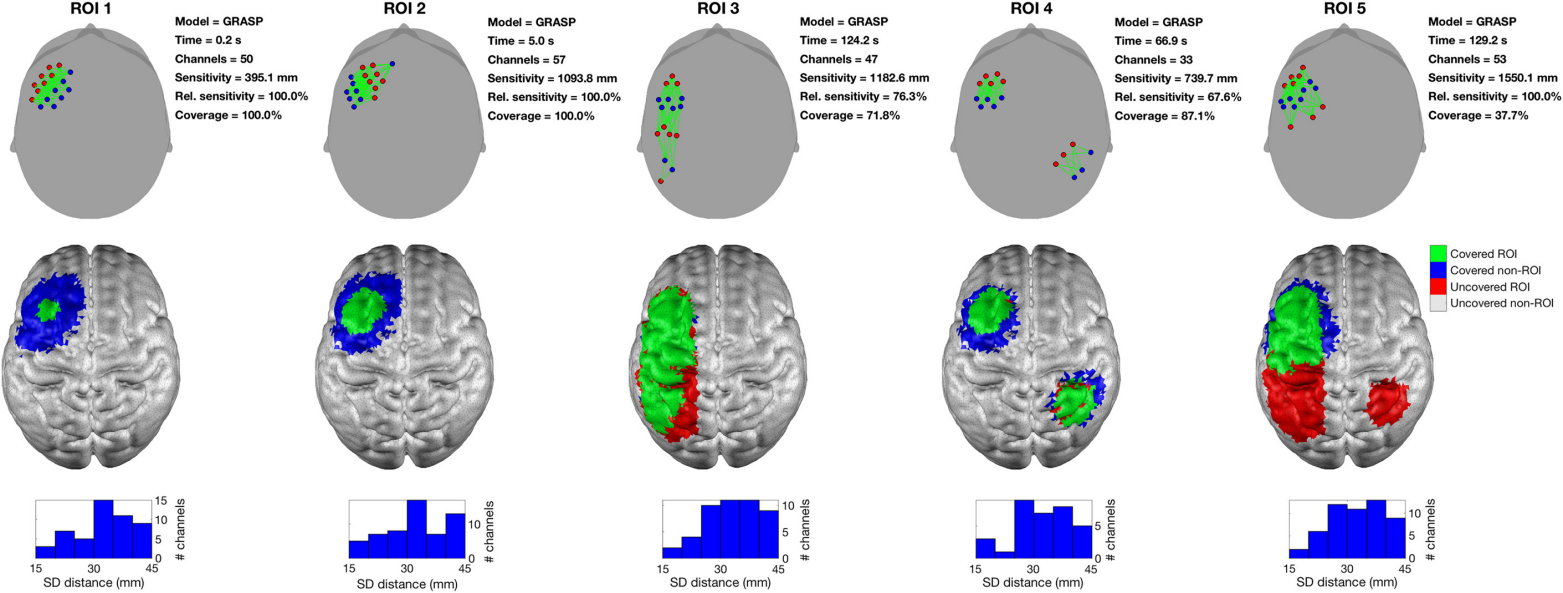
Solutions produced by Array Designer. This example shows the arrays obtained by solving the array design problem with the GRASP algorithm for all five ROIs and nS=nD=8 and cW=1. In the upper row, sources are shown as red circles, detectors as blue circles, and channels in green. For each array, the time taken, the number of viable channels, the absolute sensitivity of the array over the ROI, the relative sensitivity of the array over the ROI, and the relative coverage of the array are reported. The middle row displays the results on the GM surface. The area of the ROI covered by each array is shown in green, while any portion of an ROI that remains uncovered is shown in red. Brain areas that are covered but are outside of the ROI are shown in blue. The bottom row provides a histogram of the source–detector distances present within each array. Note that the GRASP algorithm promotes the spreading of the array solution over the ROI and is able to cover noncontiguous regions (as compared to Fig. 6). It is not able to cover ROI 5, likely because there are simply too few optodes.

**Fig. 11:**
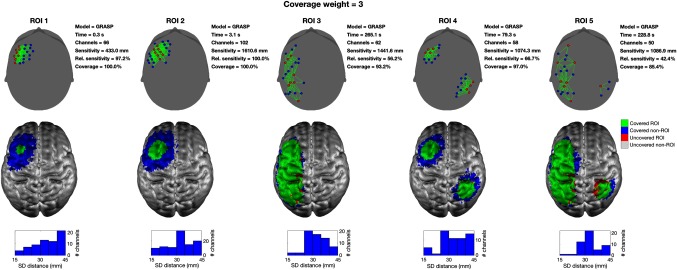
Solution produced by Array Designer. In this example are depicted the arrays obtained by solving the array design problem with the GRASP algorithm for all five ROIs and using nS=8, nD=16, and cW=3. In the upper row, sources are shown as red circles, detectors as blue circles and channels in green. For each array, the time taken, the number of viable channels, the absolute sensitivity of the array over the ROI, the relative sensitivity of the array over the ROI, and the relative coverage of the array are reported. The middle row displays the results on the GM surface. The area of the ROI covered by each array is shown in green, while any portion of an ROI that remains uncovered is shown in red. Brain areas that are covered but are outside of the ROI are shown in blue. The bottom row provides a histogram of the source–detector distances present within each array. Note here that Array Designer is able to provide good coverage of each ROI. Also note the prevalence of solutions, where dual rows of sources and dual rows of detectors are created in adjacent lines or arcs.

Figure 10 shows a series of example array solutions, for the five different ROIs, obtained with Array Designer and cW=1. The GRASP algorithm promotes the spreading of the array over the ROI and is even able to build solutions for noncontiguous regions. These results should be compared to those achieved by ${\mathrm{MIP}}_{\mathrm{MACH}}$ and depicted in Fig. 6. Figure 11 shows further examples of Array Designer’s solutions, in this case with cW=3. Note in both figures the prevalence of solutions that include dual rows of sources and dual rows of detectors arranged in adjacent lines and arcs. These “super-row” designs are evidently common among optimized array solutions, despite the fact that they have never previously been proposed as an appropriate array layout solution (at least not to our knowledge).

### 3.4 Effect of Coverage Weight (cW)

**Fig. 12:**
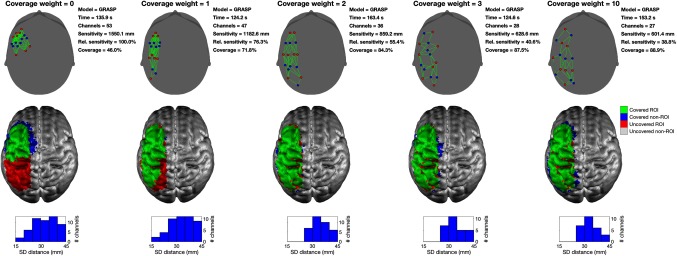
The *cW* effect. In this example are depicted the arrays obtained by solving the Array Designer problem with GRASP for ROI 3, using nS=nD=8, and varying cW. In the upper row, sources are shown as red circles, detectors as blue circles, and channels in green. For each array, the time taken, the number of viable channels, the absolute sensitivity of the array over the ROI, the relative sensitivity of the array over the ROI, and the relative coverage of the array are reported. The middle row displays the results on the GM surface. The area of the ROI covered by each array is shown in green, while any portion of an ROI that remains uncovered is shown in red. Brain areas that are covered but are outside of the ROI are shown in blue. The bottom row provides a histogram of the source–detector distances present within each array.

The *cW* parameter allows the user to define the relative importance of the coverage component of the objective function. Figure 12 shows the effect of varying cW on the array solutions produced using Array Designer for a single example ROI (ROI 3). Setting *cW* to zero is equivalent to solving the same formulation proposed by Machado et al.,^25^ and results in a clustered, focal array over the shallowest cortical region within the ROI. This array covers less than 50% of the nodes in the ROI. Setting cW=1 increases the coverage to 72%, and higher values of *cW* further increase the proportion of ROI nodes that are covered. With *cW* set to 10, the coverage approaches 90%. Note that because of the convoluted nature of the human cortex, there are likely to be deep cortical nodes in almost all ROIs, and thus achieving a coverage of 100% is likely to be rare.

## 4 Discussion

The design of an array layout is one of the first steps a researcher must undertake before starting an fNIRS study. Some fNIRS devices do offer fixed, predefined optode positions, but because the number of available optodes is almost always limited, and because the brain ROIs are study-specific, these fixed arrays are rarely ideal. As a result, many fNIRS researchers prefer to design their own arrays, tailored to their specific studies. In this paper, we have presented an approach that we believe will allow this array design step to become fully automated. Array Designer will save researchers time and effort and is highly likely to improve data quality and promote experimental standardization.

The GRASP algorithm that underpins Array Designer performs extremely well when compared to the benchmark MIP formulation ${\mathrm{MIP}}_{\mathrm{NEW}}$, and (unlike both MIP approaches) does not require specific third-party software. ${\mathrm{MIP}}_{\mathrm{MACH}}$ was originally designed by Machado et al.^25^ to produce arrays that optimally cover focal ROIs, which were the source of epileptic discharges in adult patients. Our results confirm that ${\mathrm{MIP}}_{\mathrm{MACH}}$ is very effective in covering focal ROIs but fails to provide an appropriate solution when the ROI is extended or consists of multiple noncontiguous regions. The objective function employed by both ${\mathrm{MIP}}_{\mathrm{NEW}}$ and Array Designer, which accounts for both the total sensitivity and the coverage provided by an array, is generalizable, i.e., it is applicable to any fNIRS array design problem. Because *cW* is a user-defined input, each researcher can decide whether coverage is or is not an important feature for their study and employ Array Designer to meet their requirements: if dense coverage of a small ROI is required, the user can set cW to 0, and they will obtain solutions similar to ${\mathrm{MIP}}_{\mathrm{MACH}}$; if a large ROI must be covered, *cW* should be set to a higher value to promote the spreading of the array over the ROI.

Once we had demonstrated that ${\mathrm{MIP}}_{\mathrm{NEW}}$ performs comparably to ${\mathrm{MIP}}_{\mathrm{MACH}}$ when cW=0, we were able to use ${\mathrm{MIP}}_{\mathrm{NEW}}$ as a benchmark for the performance of the Array Designer algorithm across a full range of array design simulations. The results of Fig. 9 demonstrate that the two algorithms perform comparably, with Array Designer providing marginally superior objective values on average while also having the great advantage of being much faster than ${\mathrm{MIP}}_{\mathrm{NEW}}$: an essential feature when a tool is designed to be practical for fNIRS users.

Importantly, Array Designer also easily outperforms the manually constructed, single-distance arrays that were built to emulate the standard grid or star-like arrays employed in many fNIRS studies. Array Designer always achieves higher sensitivity to the ROI compared with these manual single-distance arrays and always achieves both higher sensitivity and coverage when *cW* is set to a high value to promote the spreading of the array. These results also highlight the complexity of the array design problem: they show that it is very difficult for a user to manually design an array that is optimized without exploiting information coming from the asymmetric anatomy of the human brain and from the sensitivity distribution of a given channel.

In this paper, we have established the methodology and data flow that underpin Array Designer. Our goal now is to move toward the release of Array Designer as an easy-to-use, open-source toolbox for fNIRS users. Our hope is that this tool will soon be integrated into the Homer2/AtlasViewer fNIRS analysis package. We envisage the workflow as follows. First, a user will select a head model: this could be an adult atlas, such as the MNI152 atlas^27^ that we have used here or the Colin27 atlas^40^ available in AtlasViewer,^21^ an infant/child atlas or even an individual’s head model derived from a participant’s MRI. If the selected head model is already part of the toolbox, the precomputed PMDFs can simply be loaded. If not, a one-time-only process of PMDF computation will be performed, and the results saved for future use. Second, users will be asked to select their preferred solution space. In this study, we have used the 10-2.5 system, which we believe is the maximum density solution space that can reasonably be expected to be precisely transferred into a real-world array. As the computational challenge of array design increases exponentially with the size of the solution space, the 10-2.5 space that we selected for this investigation represents a likely worst-case problem, and thus an array design algorithm that is successful in this solution space is likely generalizable to any other. The fixed nature of the head caps associated with some commercial fNIRS systems means that some users may wish to employ a vastly smaller solution space to match their system. There is no reason to believe Array Designer would not perform very effectively in this scenario, as such problems will be easier to solve than those presented here. In the third step, the user will input the parameters that define the problem at hand: the number of available sources and detectors (*nS* and *nD*), the minimum physical distance between any two optodes (*minRhoOpt*), the maximum source–detector distance that reliably produces a good signal-to-noise with their system (*maxGoodRho*), the minimum and maximum source–detector distances allowed for a viable channel (*minRho* and *maxRho*, respectively) and the coverage weight value (*cW*). Finally, the user will be asked to define the ROI they wish to target. This last step could be performed via a number of different approaches, including: (a) building the ROIs by selecting one or more parcellated brain regions, created (for example) using automated anatomical labeling^41^ software; (b) entering the MNI coordinates of the brain ROIs and a radius to indicate the size of the ROI(s) centered over those coordinates; or (c) manually selecting the ROIs over the brain surface using a point-and-click approach. Once this information has been provided, Array Designer will be run and will output an optimized array solution. Each optode that forms part of the array solution will be associated with a 10-2.5 label (or equivalent if a different solution space is selected), to make it as easy as possible for users to relate the output of Array Designer to their actual fNIRS hardware. Every Array Designer solution will also include associated array quality measures, specifically the total sensitivity of the optimized array design over the ROI (in mm), the percentage of the ROI covered by that array and the average, minimum, and maximum source–detector separation of its channels. Although AtlasViewer can provide qualitative comparisons of array quality, no fNIRS study to date has (to our knowledge) reported any quantified measure of “array quality” as part of its experimental methods. The ability to provide these (particularly in some standard, parcellated brain spaces) would significantly improve interstudy comparisons and would likely improve standardization across the fNIRS field. Ideally, each fNIRS paper would specify their defined ROI and their array’s sensitivity to and coverage of that ROI, as well as the range of source–detector separations employed [e.g., adult MNI152 model, left precentral gyrus, 2000 mm, 85%, SD separation mean (range): 35 mm, (25 to 50 mm)], so that array quality can be compared from problem to problem. These metrics are easy to understand and compare, and could easily be computed for manually designed arrays. One could even quote these metrics on a subject-by-subject basis in a way that accounts for excluded or noisy channels.

It is worth noting that the brain template we employed is asymmetric. As such, the arrays designed by Array Designer will likely also be asymmetric between hemispheres. Given the natural asymmetry of the human brain that is evident in our selected atlas, we feel it is correct to design bilateral arrays in one step and accept their asymmetry. However, given that individual variability might far exceed the subtle asymmetry of the average brain template, users may prefer to use Array Designer to produce symmetric arrays. A “force symmetry” option, which will force solutions to maintain hemispheric symmetry, can be easily incorporated into the framework of Array Designer, and is an option we expect to provide in the future.

Array Designer does have several important limitations. First, Array Designer considers surface PMDFs rather than volumetric PMDFs: depth information is therefore somewhat neglected. While this is a valid assumption when the goal is to design fNIRS arrays, depth sensitivity is an essential element when designing diffuse optical tomography (DOT) arrays. However, future amendments to Array Designer could well incorporate fully 3-D PMDFs, which would allow users to specify a volume of interest and design appropriate DOT arrays to sample that volume. We believe these modifications will be relatively straightforward, given the framework of Array Designer established here. Second, Array Designer does not take into account the need for short-separation channels:^4^^,^^42^ we recommend that users wishing to include short-separation channels in their array should simply add these to the array solutions provided by Array Designer in a manner that meets the requirements of their device. Third, while the algorithm performs exceptionally well when compared to the MIP formulations, the GRASP algorithm provides no guarantee that the solution it provides is the optimum solution. When large arrays and large ROIs are employed, it is in fact likely that better solutions do exist (for context, the relatively simple case depicted in the first column of Figs. 4, 6, and 10 has ∼200 billion possible solutions). An advantage of the MIP approaches is that they provide a measure of how far a given solution is likely to be from the true optimum solution (the bounds described above); GRASP does not provide this type of information. However, while Array Designer may not tell users how far they are from an optimum solution, the superior results achieved by GRASP relative to ${\mathrm{MIP}}_{\mathrm{NEW}}$ suggest that users can be relatively confident that any solution provided by Array Designer is likely to be the best currently achievable through any method.

In this paper, we have described a generalizable methodological framework for the automated design of fNIRS arrays. Array Designer works effectively in large solution spaces, on standard consumer-grade computers, requires limited user inputs that are easy to define and understand, and provides quantified array quality metrics that can be compared across subjects, experiments and groups. By developing Array Designer in to an easy-to-use toolbox, we hope to redefine the way in which fNIRS arrays are designed: saving researchers’ time, improving their data quality by increasing cortical sensitivity, and promoting experimental standardization.

## Appendix

In this section, the formulation of the MIP approaches is detailed. The following notation will be employed:

• P: set of possible scalp positions for optode placement
• V: set of all nodes
• R⊆V: subsets of nodes included in the ROI
• $I_{1}\subseteq P\times P$: set of incompatible pairs of positions because of the *minRhoOpt* distance constraints between any two optodes
• $I_{2}\subseteq P\times P$: set of incompatible pairs of positions because of the *minRho* and *maxRho* distance constraints between source and detectors.
• $a_{pq}^{v}$: sensitivity value at node v for channel composed by source p and detector q. This is equivalent to ${\mathrm{PMDF}}_{i}(n)$ of Eq. (2).

### A.1 ${\mathrm{MIP}}_{\mathrm{MACH}}$

This model defines the following variables and parameters:

$$\;x_{p}=\{\begin{array}{ll} 1, & \text{if a source is placed in position}\;p\\ 0, & \text{otherwise} \end{array},$$ (4)

$$y_{q}=\{\begin{array}{ll} 1, & \text{if a detector is placed in position}\;q\\ 0, & \text{otherwise} \end{array},$$ (5)

$$b_{pq}=\underset{v\in R}{\sum }a_{pq}^{v},$$ (6)

$$w_{q}=\{\begin{array}{ll} \underset{p\in P}{\sum }b_{pq}x_{p}, & \text{if}\;y_{q}=1\\ 0, & \text{otherwise} \end{array}.$$ (7)

The model uses binary variables (x and y) to encode the main decisions of where to place optodes on the scalp and artificial continuous variables w to correctly evaluate objective function. The model reads as follows:

$$\max\;\underset{q\in P}{\sum }w_{q},$$ (8)

with the following constraints:

$$\underset{p\in P}{\sum }x_{p}=nS,$$ (9)

$$\sum_{q\in P}y_{q}=nD,$$ (10)

$$x_{p}+y_{p}\leq 1\;\forall \;p\in P,$$ (11)

$$w_{q}\leq My_{q}\;\forall \;q\in P,$$ (12)

$$w_{q}\leq \underset{p\in P}{\sum }b_{pq}x_{p}\;\forall \;q\in P,$$ (13)

$$x_{i}+x_{j}\leq 1\;\forall \;(i,j)\in I_{1},$$ (14)

$$y_{i}+y_{j}\leq 1\;\forall \;(i,j)\in I_{1},$$ (15)

$$x_{p}+y_{q}\leq 1\;\forall \;(p,q)\in I_{2},$$ (16)

$$x_{p}\in \{0,1\}\;\forall \;p\in P,$$ (17)

$$y_{q}\in \{0,1\}\;\forall \;q\in P,$$ (18)

$$w_{q}\geq 0\;\forall \;q\in P,$$ (19)

where M is a so-called big-M, i.e., a suitable large enough coefficient. Constraint in Eq. (9) [Eq. (10)] makes sure that the required number of sources (detectors) is placed, while constraint in Eq. (11) encodes the fact that a source and a detector cannot be placed in the same position. Constraints in Eqs. (12) and (13) are a linear encoding of Eq. (7), and constraints in Eqs. (14)–(16) encode the incompatibilities between optode positions based on distance constraints. Because of the big-M constraint linking variables w with the binary variables y, the linear programming relaxation is usually rather weak in this model.

### A.2 ${\mathrm{MIP}}_{\mathrm{NEW}}$

First, we will describe the ${\mathrm{MIP}}_{\mathrm{NEW}}$ formulation when using cW=0, and then the one employed when cW>0. ${\mathrm{MIP}}_{\mathrm{NEW}}$ does not use continuous variables w, but introduces binary variables $z_{pq}=x_{p}y_{q}$, one for each possible channel.

The model reads as follows:

$$\max\underset{p\in P}{\sum }\underset{q\in P}{\sum }b_{pq}z_{pq},$$ (20)

$$\underset{p\in P}{\sum }x_{p}=nS,$$ (21)

$$\underset{q\in P}{\sum }y_{q}=nD,$$ (22)

$$x_{p}+y_{p}\leq 1\;\forall \;p\in P,$$ (23)

$$\underset{q\in P}{\sum }z_{pq}=nDx_{p}\;\forall \;p\in P,$$ (24)

$$\underset{p\in P}{\sum }z_{pq}=nSy_{q}\;\forall \;q\in P,$$ (25)

$$x_{i}+x_{j}\leq 1\;\forall \;(i,j)\in I_{1},$$ (26)

$$y_{i}+y_{j}\leq 1\;\forall \;(i,j)\in I_{1},$$ (27)

$$x_{p}+y_{q}\leq 1\;\forall \;(p,q)\in I_{2},$$ (28)

$$x_{p}\in \{0,1\}\;\forall \;p\in P,$$ (29)

$$y_{q}\in \{0,1\}\;\forall \;q\in P,$$ (30)

$$z_{pq}\in \{0,1\}\;\forall \;p,q\in P.$$ (31)

Most constraints have the same meaning as in the previous formulation. Constraints in Eqs. (24) and (25) link variables $z_{pq}$ to variables x, y, and encode the definition $z_{pq}=x_{p}y_{q}$. Furthermore, the constraints in this new model are very combinatorial, with sensitivity coefficients only appearing in the objective. MIP solvers are usually tailored to exploit these structures. This model uses the same precomputed $b_{pq}$, as there is no need to compute the sensitivity at each individual voxel yet. Note that there is no need to explicitly linearize the products $z_{pq}=x_{p}y_{q}$, as this is effectively taken care of by constraints in Eqs. (24) and (25).

Furthermore, the LP relaxation can be strengthened by adding the following family of valid inequalities:

$$z_{pq}\leq x_{p}\;\forall \;p,q\in P,$$ (32)

$$z_{pq}\leq y_{q}\;\forall \;p,q\in P.$$ (33)

However, those should not be added directly to the model, as they slow down the solution of the LP relaxation significantly, but rather separated on the fly when needed. In our implementation, we just declared them as user cuts, letting the MIP solver to deal with them in the most efficient way.

When taking coverage into account, the model presented above can be easily extended to deal with the individual node sensitivities by adding the following variables:

$$s_{v}=\sum_{p\in P}\sum_{q\in P}a_{pq}^{v}z_{pq},$$ (34)

$$c_{v}=\{\begin{array}{ll} 1, & \text{if node}\;v\;\text{is covered}\\ 0, & \text{otherwise} \end{array}.$$ (35)

Note that binary variable c is used for counting the number of covered voxels, whereas continuous variable s encodes the sensitivity at each node of the ROI.

The model thus reads as follows:

$$\max\;\frac{1}{S_{\max}}\underset{v\in R}{\sum }s_{v}+\frac{cW}{\left|R\right|}\underset{v\in R}{\sum }c_{v},$$ (36)

$$\underset{p\in P}{\sum }x_{p}=nS,$$ (37)

$$\underset{q\in P}{\sum }y_{q}=nD,$$ (38)

$$x_{p}+y_{p}\leq 1\;\forall \;p\in P,$$ (39)

$$\underset{q\in P}{\sum }z_{pq}=nDx_{p}\;\forall \;p\in P,$$ (40)

$$\underset{p\in P}{\sum }z_{pq}=nSy_{q}\;\forall \;q\in P,$$ (41)

$$s_{v}=\underset{p\in P}{\sum }\underset{q\in P}{\sum }a_{pq}^{v}z_{pq}\;\forall \;v\in R,$$ (42)

$$s_{v}\geq C_{\mathrm{thresh}}c_{v}\;\forall \;v\in R,$$ (43)

$$x_{i}+x_{j}\leq 1\;\forall \;(i,j)\in I_{1},$$ (44)

$$y_{i}+y_{j}\leq 1\;\forall \;(i,j)\in I_{1},$$ (45)

$$x_{p}+y_{q}\leq 1\;\forall \;(p,q)\in I_{2},$$ (46)

$$x_{p}\in \{0,1\}\;\forall \;p\in P,$$ (47)

$$y_{q}\in \{0,1\}\;\forall \;q\in P,$$ (48)

$$z_{pq}\in \{0,1\}\;\forall \;p,q\in P,$$ (49)

$$s_{v}\geq 0\;\forall \;v\in R,$$ (50)

$$c_{v}\in \{0,1\}\;\forall \;v\in R.$$ (51)

Equation (36) coincides with Eq. (1). Constraint in Eq. (42) encodes the definition in Eq. (34) of the s variable, and constraint in Eq. (43) makes sure that the binary variable $c_{v}$ can be set to one, and thus counts the node as covered, only if the signal at that node is above the threshold. Differently from the previous model, this model cannot use the precomputed $b_{pq}$ quantity, and $a_{pq}^{v}$ has to be used directly instead. In addition to a number of variables quadratic in the number of possible positions on which to put an optode, this new model also needs two further variables (one binary and one continuous) for each node in the ROI. Finally, the LP relaxation can be strengthened with the same family of valid inequalities described for the previous model.
